# Analysis of risk factors and their predictive efficacy for postoperative gastrointestinal dysfunction in patients with severe traumatic brain injury

**DOI:** 10.3389/fneur.2025.1614833

**Published:** 2025-09-17

**Authors:** Caiyun Yang, Chunyang Xu, Shun Wen, Sun Yu, Liang Yang, Liming Pan, Feng Xu

**Affiliations:** ^1^Department of Emergency Medicine, The First Affiliated Hospital of Soochow University, Suzhou, China; ^2^Department of Emergency Medicine, Changshu Hospital Affiliated to Soochow University, The First People’s Hospital of Changshu, Changshu, China

**Keywords:** traumatic brain injury, gastrointestinal dysfunction, risk factors, precision medicine, warning effectiveness

## Abstract

**Introduction:**

To investigate the risk factors and their warning effectiveness for postoperative gastrointestinal dysfunction in patients with severe traumatic brain injury (sTBI).

**Methods:**

A total of 160 patients with sTBI were divided into the gastrointestinal dysfunction group (*n* = 64) and the non-gastrointestinal dysfunction group (*n* = 96). Data including gender, age, past medical history, types of intracranial hematoma, injury type, preoperative GCS, postoperative GCS, postoperative intracranial pressure (ICP), operation time, intraoperative blood pressure, enteral nutrition initiation time, APACHE II score, SOFA score, insulin-like growth factor 1 (IGF-1), and fecal calprotectin (FC), were collected. Univariate and multivariate binary logistic regression analyses were conducted. The ROC curve and AUC were plotted to assess the predictive efficacy of each risk factor for gastrointestinal dysfunction.

**Results:**

The univariate analysis revealed that intraoperative blood pressure, past medical history, postoperative ICP, enteral nutrition initiation time, postoperative GCS score, neuron specific enolase, S100, IGF-1, and FC were significantly associated with the occurrence of gastrointestinal dysfunction. In addition, multivariate binary logistics regression analysis indicated that IGF-1, postoperative ICP, and FC were significantly associated with postoperative gastrointestinal dysfunction. Among these factors, the ROC curve of IGF-1 demonstrated a higher warning efficacy, which was similar to FC, whereas the ROC curve of ICP showed slightly lower AUC.

**Discussion:**

IGF-1, postoperative ICP, and FC are independent factors affecting postoperative gastrointestinal dysfunction in patients with sTBI. Notably, IGF-1 and FC exhibit higher predictive efficacy, while postoperative ICP shows a slightly lower predictive efficacy.

## Introduction

Traumatic brain injury is a significant global public health issue, contributing to high rates of morbidity, mortality, and long-term disability. Severe traumatic brain injury (sTBI), defined as a Glasgow Coma Score (GCS) ≤ 8, is particularly devastating, with mortality rates ranging between 30 and 40% (1, 2). Advances in surgical and critical care management have improved survival rates for sTBI patients. However, these patients are often burdened with secondary complications that impair recovery and quality of life. Among these complications, postoperative gastrointestinal dysfunction is a frequent yet underappreciated issue that can severely impact clinical outcomes.

Postoperative gastrointestinal dysfunction in sTBI patients manifests in various forms, including stress ulcers, gastrointestinal bleeding caused by ischemia of the gastrointestinal mucosa (3), abdominal distension, diarrhea due to impaired gastrointestinal motility, gastric retention, and disruption of the intestinal barrier (4). These complications are not merely localized issues but have systemic consequences. Emerging evidence highlights the bidirectional relationship between the brain and gut, known as the brain-gut axis, which plays a critical role in the pathophysiology of gastrointestinal dysfunction in sTBI patients. Specifically, gastrointestinal dysfunction can exacerbate brain injury through pathways involving inflammatory mediators (5, 6), endocrine signals (7), and alterations in gut microbiota (8). Such interactions may worsen systemic inflammation, impair nutrient absorption, and contribute to the development of multiple organ dysfunction syndrome, ultimately increasing mortality rates (9).

Despite the recognized importance of gastrointestinal dysfunction in critically ill patients, there is limited understanding of the specific risk factors that predispose sTBI patients to postoperative gastrointestinal dysfunction. Furthermore, while some biomarkers, such as fecal calprotectin (FC) and insulin-like growth factor 1 (IGF-1), have been implicated in gastrointestinal inflammation and mucosal barrier integrity, their roles in predicting gastrointestinal dysfunction in sTBI patients have not been systematically explored.

The novelty of this study lies in its comprehensive approach to identifying and evaluating risk factors for postoperative gastrointestinal dysfunction in sTBI patients. On integrating clinical parameters (e.g., intracranial pressure [ICP], GCS scores, and timing of enteral nutrition) with biomarker analysis (e.g., IGF-1 and FC), this study aims to provide a multifaceted perspective of the predictors and underlying mechanisms of gastrointestinal dysfunction in this high-risk population. In addition, the predictive efficacy of these factors, assessed through receiver operating characteristic (ROC) curve analysis, offers valuable insights into their potential for early warning and targeted intervention. The findings from this study are expected to guide clinical strategies aimed at early prevention and management of postoperative gastrointestinal dysfunction, ultimately improving outcomes for sTBI patients.

## Materials and methods

### Study cohort description

This retrospective cohort study included 160 patients diagnosed with sTBI who underwent surgical intervention and were admitted to Changshu Hospital Affiliated with Soochow University between January 5, 2021 and December 20, 2023. Among the participants, 107 were male and 53 were female, with ages ranging from 21 to 81 years (mean age: 58.4 ± 14.2 years). Patients were categorized into two groups based on the presence or absence of gastrointestinal dysfunction within 7–10 days following admission: the gastrointestinal dysfunction group (*n* = 64) and the non-gastrointestinal dysfunction group (*n* = 96).

Gastrointestinal dysfunction was defined according to the 2012 criteria established by the European Society of Intensive Care Medicine’s Working Group on Abdominal Diseases, which describes acute gastrointestinal injury as gastrointestinal dysfunction caused by the acute illness of critically ill patients. In this study, patients classified as severity levels II to IV were included in the gastrointestinal dysfunction group.

All patients underwent surgical treatment, which included procedures such as craniotomy for hematoma evacuation, ventriculostomy, craniectomy for decompression, and placement of a cerebral pressure probe. Intracranial pressure (ICP) monitoring was performed in all surgical patients using a cerebral ICP probe.

#### Inclusion criteria

- A documented history of head trauma confirmed by head CT scan indicating brain injury, with a GCS score of 3 to 8.
- Age ≥ 18 years.
- Surgical treatment for sTBI.

#### Exclusion criteria

- Presence of thoracoabdominal injuries or other severe injuries requiring surgery.
- Death or withdrawal from treatment within 1 week of admission.
- Pre-existing digestive tract diseases.
- Severe comorbidities, including heart failure, liver or kidney dysfunction, malignant tumors, or active infections.

### Observational indicators

The following data were collected for each patient:

- Demographic information: Gender, age, and past medical history (e.g., hypertension, diabetes, immunosuppression, coronary heart disease).
- Clinical characteristics: Types of intracranial hematoma (subdural hematoma, extradural hematoma, intracerebral hematoma), types of injury (e.g., traffic accident, fall from height), preoperative GCS, postoperative GCS, postoperative ICP, surgery duration, intraoperative blood pressure, and time of enteral nutrition initiation.
- Scoring systems: Acute Physiology and Chronic Health Evaluation II (APACHE II) score and Sequential Organ Failure Assessment (SAPS II) score.
- Biomarkers: IGF-1 and FC: Blood samples for IGF-1 measurement were collected at the time of admission (within 24 h) and again at 7 days post-surgery. Fecal samples for FC analysis were collected on the day of ICU admission and at day 7 post-surgery.

#### Sample collection and processing

Blood samples for IGF-1 were drawn in a fasting state (at least 8 h of fasting) and were immediately processed by centrifugation at 4 °C for 10 min. The serum was then stored at −80 °C until analysis. Fecal samples for FC measurement were collected in sterile containers, processed, and stored at −20 °C until analysis. Both biomarkers were quantified using standard commercial assay kits (e.g., enzyme-linked immunosorbent assay [ELISA] for IGF-1 and calprotectin for FC), according to the manufacturer’s instructions.

#### Time of enteral nutrition initiation

The timing of the initiation of enteral nutrition was recorded. Additionally, the adequacy of enteral nutrition, defined as the proportion of the target daily caloric intake that was actually provided, was monitored to ensure that patients received the recommended amount of nutrition according to clinical guidelines. Patients who did not meet the target nutritional goals despite early initiation were identified and analyzed for potential impacts on outcomes.

To identify risk factors for postoperative gastrointestinal dysfunction, single-factor and multivariate binary logistic regression analyses were conducted. The predictive efficacy of each risk factor was evaluated using receiver operating characteristic (ROC) curves, and the area under the curve (AUC) was calculated.

### Statistical analysis

Statistical analysis was performed using SPSS version 26.0. The Kolmogorov–Smirnov test was used to assess data normality. Continuous variables that followed a normal distribution were presented as *x* ± *s* and compared between groups using the two-sample *t*-test. Non-normally distributed data were expressed as medians (M) with interquartile ranges (Q1, Q3) and compared using the Mann–Whitney *U* test. Categorical variables were presented as frequencies and percentages, and comparisons were made using the *χ*^2^ test.

To address potential multicollinearity among predictor variables, the variance inflation factor (VIF) was calculated for each variable included in the multivariate logistic regression analysis. A VIF value greater than 10 was considered indicative of significant multicollinearity. The results of the multicollinearity analysis were considered when interpreting the regression coefficients. To assess the goodness-of-fit of the multivariate logistic regression model, the Hosmer–Lemeshow test was performed. This test evaluates whether the observed event rates match the expected event rates in subgroups of the model. A *p*-value > 0.05 suggests a good fit. Additionally, the Nagelkerke *R*^2^ value was reported to indicate the proportion of variance in the outcome variable explained by the model. A higher *R*^2^ value indicates a better explanatory power of the model.

Variables with a significance level of *p* < 0.05 in the univariate analysis were included in the multivariate logistic regression analysis to identify independent risk factors. The ROC curve and AUC were used to evaluate the predictive performance of each significant risk factor. All statistical tests were two-sided, and a *p*-value < 0.05 was considered statistically significant.

### Ethical considerations

This study was conducted in accordance with the principles outlined in the Declaration of Helsinki. Written informed consent was obtained from the families of all participants after they were informed about the study’s objectives and assured of privacy and confidentiality. The study protocol was reviewed and approved by the Medical Ethics Committee of The First Affiliated Hospital of Soochow University on October 10, 2020 (Approval No.: L2020015).

## Results

### Analysis of single factors for postoperative gastrointestinal dysfunction in sTBI patients

Univariate analysis showed that intraoperative blood pressure, past medical history, postoperative ICP, enteral nutrition initiation time, postoperative GCS score, NSE, S100, IGF-1, and FC were associated with postoperative gastrointestinal dysfunction in patients with sTBI (*p* < 0.05). In contrast, gender, age, type of injury, location of brain injury, operation time, admission GCS score, APACHE II, and SAPS II were not associated with the occurrence of gastrointestinal dysfunction (*p* > 0.05) (Table 1).

**Table 1:** Univariate analysis of postoperative gastrointestinal dysfunction in patients with sTBI.

Variable	Gastrointestinal dysfunction group (*n* = 64)	Non-gastrointestinal dysfunction group (*n* = 96)	*X*^2^/*z*/*t*	*p*-value
Gender				
Male	39(60.9%)	68(70.8%)	1.69	0.231
Female	25(39.1%)	28(29.2%)		
Age (years)	61.47 ± 15.74	56.59 ± 15.31	−1.946	0.053
Type of injury				
Traffic accident	39(60.9%)	53(53.2%)	0.536	0.765
Fall from height	6(9.4%)	11(11.5%)		
Other	19(29.7%)	32(33.3%)		
Site of brain injury				
Epidural	21(32.8%)	37(38.5%)	0.625	0.731
Subdural hematoma	31(48.4%)	44(45.8%)		
Cerebral lobe hematoma	12(18.8%)	15(15.6%)		
Intraoperative blood pressure (mmHg)				
High blood pressure (SBP > 140 mmHg)	3(4.7%)	23(23.9%)	78.03	<0.001*
Low blood pressure (SBP < 100 mmHg)	22(34.4%)	28(29.2%)		
Fluctuation of blood pressure (SBP fluctuated by more than 50 mmHg)	36(56.2%)	2(2.1%)		
Blood pressure is stable	3(4.7%)	43(44.8%)		
Past medical history				
None	42(65.6%)	80(83.3%)	6.649	0.01*
Present (hypertension, diabetes, immunosuppression, or coronary heart disease)	22(34.4%)	16(16.7%)		
Postoperative ICP (mmHg)	16(14.5,20)	8(5,10)	−8.98	<0.001*
Surgery duration (hours)	3.1(2.9,3.6)	3.2(2.3,4.8)	−1.566	0.117
Time of enteral nutrition initiation (hours)	20.65 ± 4.23	27.2 ± 8.22	−6.8	<0.001*
Preoperative GCS	5(4,5)	4(4,6)	−1.910	0.056
Postoperative GCS	4(3,4)	5(3,6)	−2.907	0.04*
NSE (ng/ml)	78.84(45.88,109.21)	34.54(24.87,70.96)	−4.709	<0.001*
S100 (μg/ml)	1.28(1.02,1.53)	0.35(0.24,0.43)	−8.852	<0.001*
APACHE II	19.5(16,25)	19(14,26)	−0.274	0.784
SAPS II	45.55 ± 20.4	45.21 ± 18.3	0.105	0.916
IGF-1 (ng/ml)	43.32 ± 17.2	94.92 ± 14.4	19.63	<0.001*
FC (beats per minute)	35.71 ± 16.48	60.23 ± 23.44	−9.187	<0.001*

*p < 0.05.

### Analysis of multiple factors for postoperative gastrointestinal dysfunction in patients with sTBI using a binary logistic regression model

The results of the multiple-factor binary logistic regression analysis indicated that IGF-1, postoperative ICP, and FC were significantly associated with the development of postoperative gastrointestinal dysfunction in patients with sTBI (*p* < 0.05). However, intraoperative blood pressure, postoperative GCS, past medical history, NSE, S100, and the time of enteral nutrition initiation showed no significant association with the occurrence of gastrointestinal dysfunction (*p* > 0.05) (Table 2). The goodness-of-fit of the multivariate logistic regression model was evaluated using the Hosmer-Lemeshow test, which yielded a *p*-value of 0.712, suggesting that the model had a good fit. Additionally, the Nagelkerke *R*^2^ value was 0.265, indicating that 26.5% of the variance in postoperative gastrointestinal dysfunction was explained by the model.

**Table 2:** Multivariate binary logistic regression analysis of postoperative gastrointestinal dysfunction in patients with sTBI.

Variable	*Β*	SE	Wald	OR	95%CI	*P*
Intraoperative blood pressure (mmHg)	0.041	0.748	0.003	1.042	0.24 ~ 4.519	0.956
Postoperative GCS	−0.085	0.313	0.073	0.919	0.498 ~ 1.697	0.787
Past medical history	0.871	2.035	0.183	2.389	0.044 ~ 128.977	0.669
NSE (ng/mL)	−0.006	0.019	0.089	0.994	0.958 ~ 1.032	0.765
S100 (μg/mL)	4.168	1.878	4.926	64.576	0.628 ~ 2561.921	0.066
IGF-1 (ng/mL)	−0.145	0.053	7.370	0.865	0.779 ~ 0.960	0.007*
Time of enteral nutrition initiation (hours)	0.275	0.171	2.604	1.317	0.943 ~ 1.839	0.107
Postoperative ICP (mmHg)	0.326	0.124	6.946	1.385	1.087 ~ 1.765	0.008*
FC (beats per minute)	0.425	0.231	5.630	1.672	1.342 ~ 1.953	0.005*

*p < 0.05.

### Warning effectiveness of related risk factors on postoperative gastrointestinal dysfunction in patients with sTBI

**Figure 1:**
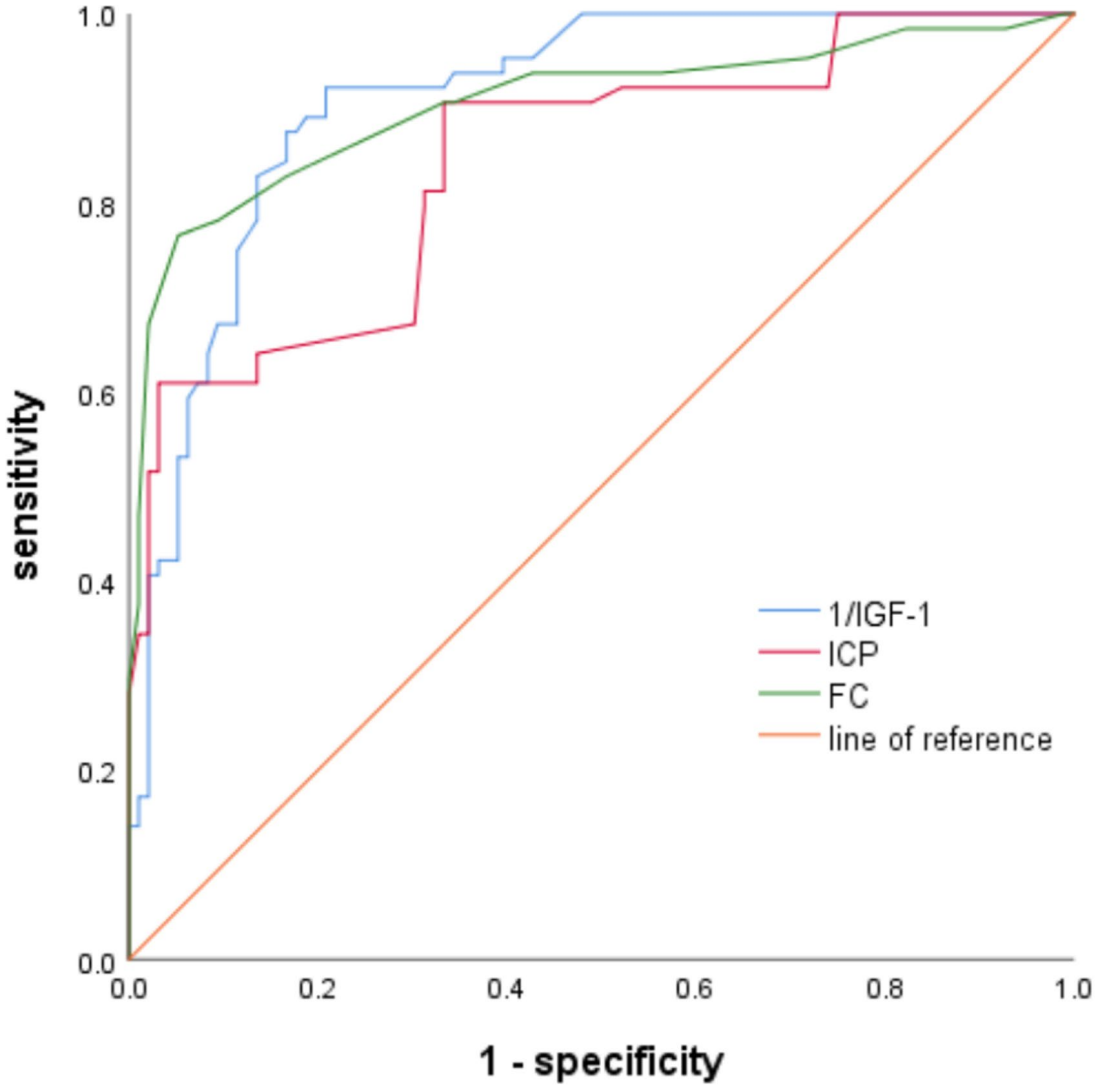
The predictive effectiveness of ROC for gastrointestinal dysfunction alerts in patients with sTBI-related risk factors.

The ROC curve was plotted to determine the predictive efficacy of the identified risk factors. The area under the ICP curve was 0.841, with a cut-off value of 16 and a sensitivity and specificity of 82.8 and 83.3%, respectively. The area under the IGF-1 curve was 0.909, with a cut-off value of 68 and a sensitivity and specificity of 87.5 and 82.3%, respectively. The area under the FC curve was 0.904, with a cut-off value of 42, and a sensitivity and specificity of 86.5 and 84.1%, respectively (Table 3 and Figure 1).

**Table 3:** Analysis of the early warning effect of relevant risk factors on the occurrence of postoperative gastrointestinal dysfunction in patients with sTBI.

Variable	AUC	Sensitivity (%)	Specificity (%)	95%CI	*P*
ICP (mmHg)	0.841	82.8	83.3	0.778 ~ 0.905	<0.001*
IGF-1 (ng/mL)	0.909	87.5	82.3	0.865 ~ 0.954	<0.001*
FC (beats per minute)	0.904	86.5	84.1	0.850 ~ 0.957	<0.001*

*p < 0.05.

## Discussion

Gastrointestinal dysfunction in critically ill patients is a significant complication that can lead to malnutrition, prolonged hospitalization, multiple organ dysfunction, and increased mortality rates (10). Previous studies have highlighted that TBI patients with gastrointestinal failure tend to have longer ICU stays and lower Glasgow Outcome Scale scores at the 3-month follow-up after discharge (11). Therefore, identifying risk factors for gastrointestinal dysfunction in sTBI patients is essential for early intervention and improved outcomes.

This study demonstrated that ICP, FC, and IGF-1 are independent risk factors for gastrointestinal dysfunction in sTBI patients, each showing significant predictive value. The findings provide critical insights into the pathophysiology of gastrointestinal dysfunction in these patients and suggest potential avenues for clinical management and preventative strategies. Although previous studies (12) have explored the role of biomarkers, such as IGF-1 and FC in gastrointestinal dysfunction, the current study presented novel insights by specifically concentrating on their predictive value in the context of sTBI. While IGF-1 has been previously linked to outcomes in neurocritical care, its role in gastrointestinal dysfunction post-TBI remains underexplored. Our findings highlight how decreased IGF-1 levels serve as an early biomarker for gastrointestinal dysfunction in sTBI patients, complementing neuroendocrine disruptions often observed in TBI cases. Similarly, FC has been well-established as an indicator of intestinal inflammation in various gastrointestinal diseases; however, its predictive value for gastrointestinal dysfunction in the neurocritical care setting is less understood. The current study demonstrated that FC could not only reflect early gastrointestinal inflammation, but also predict dysfunction even before overt clinical symptoms appear in sTBI patients. This supports the potential of FC as a non-invasive tool for early diagnosis and intervention. Furthermore, while previous research (13) has primarily studied these biomarkers in individual contexts, this study provided evidence of their combined predictive efficacy for gastrointestinal dysfunction, thus presenting a broader view of their clinical utility in a cohort of sTBI patients. Incorporating these biomarkers into clinical practice allows for more effective identification of high-risk patients, which is a significant advancement over previous studies that typically lacked a comprehensive biomarker-based approach.

### Intracranial pressure (ICP)

The ICP is a key indicator for postoperative monitoring in sTBI patients, and its role in predicting patient prognosis has been well-documented (12). Elevated ICP is associated with increased sympathetic nervous system activity, leading to systemic and gastrointestinal symptoms. Specifically, raised ICP can disrupt gastrointestinal motility, impair water and electrolyte absorption, and delay gastric emptying (13). These effects are thought to result from neuroendocrine dysregulation and compromised blood perfusion in the gastrointestinal tract caused by elevated ICP.

Studies have shown that ICP levels above 20 mmHg significantly reduce cerebral blood flow and necessitate preventive measures against secondary brain injury (12). Furthermore, prolonged periods of ICP elevation above 20–25 mmHg aggravate treatment challenges and increase mortality risk. This study corroborates these findings, demonstrating that postoperative ICP is an independent risk factor for gastrointestinal dysfunction in sTBI patients.

The mechanisms underlying this relationship may include:

Neuroendocrine disorders: Elevated ICP stimulates nerve centers such as the hypothalamus and brainstem, triggering excessive sympathetic nervous system activity and the release of hormones like catecholamines. These hormonal changes constrict gastrointestinal blood vessels, reduce blood flow, and impair gastrointestinal motility and secretion.

Brain-gut axis dysregulation: The brain-gut axis, a neuroendocrine network connecting the central nervous system and gastrointestinal tract, plays a bidirectional regulatory role. Elevated ICP disrupts this axis, transmitting stress signals to the gastrointestinal tract and impairing its motor and barrier functions.

Active management of postoperative ICP in sTBI patients is essential to mitigate gastrointestinal dysfunction, reduce complications, and improve overall outcomes.

### Fecal calprotectin (FC)

The FC is a calcium-zinc-binding protein secreted by neutrophils, macrophages, and monocytes, serving as a marker of intestinal inflammation. Elevated FC levels reflect damage to the intestinal mucosa and impaired absorption function. Studies have shown that gastrointestinal inflammation and injury result in significantly increased FC concentrations, which can be up to six times higher than calcium-binding protein levels in the bloodstream (14). FC detection is a non-invasive, simple, and stable diagnostic tool, making it a valuable indicator for early gastrointestinal inflammatory disease detection (15).

The Consensus Opinion on the Diagnosis and Treatment of Inflammatory Bowel Disease (2018·Beijing) emphasized FC’s utility in assessing the degree of intestinal mucosal inflammation (16). FC also correlates with endoscopic findings in inflammatory bowel diseases such as ulcerative colitis and Crohn’s disease (17, 18). In addition, FC is useful for differentiating organic from non-organic intestinal lesions (18).

This study identified FC as an independent risk factor for gastrointestinal dysfunction in sTBI patients. Elevated FC levels were detectable in some patients even before clinical symptoms of gastrointestinal dysfunction appeared, suggesting that intestinal inflammation may precede overt symptoms. FC’s sensitivity to early-stage inflammation highlights its potential as a predictive biomarker for gastrointestinal dysfunction, enabling timely clinical intervention.

### Insulin-like growth factor 1 (IGF-1)

IGF-1 is a polypeptide hormone with an insulin-like structure that regulates cell growth, proliferation, migration, differentiation, and apoptosis. IGF-1 receptors are widely distributed in tissues such as muscles, bones, intestines, and nerves, playing key roles in growth, development, and maintaining intestinal mucosal barrier function (19).

Following brain injury, IGF-1 expression changes both locally and in peripheral circulation. Animal studies have shown increased local IGF-1 expression in injured brain regions within 24 h of the injury, persisting for weeks (20, 21). However, serum IGF-1 levels tend to decrease after brain injury, correlating with cognitive dysfunction and poor long-term prognosis (22, 23). Higher serum IGF-1 levels have been associated with better recovery of white matter and memory function in brain injury patients (22).

IGF-1 also plays a critical role in intestinal health, where it promotes intestinal mucosal hyperplasia, improves barrier function, and reduces bacterial translocation in conditions such as severe burns and liver cirrhosis (24, 25). Exogenous IGF-1 supplementation has been demonstrated to reduce inflammation and enhance intestinal mucosal integrity in animal models of necrotizing colitis (26).

This study revealed that decreased serum IGF-1 levels are an independent risk factor for postoperative gastrointestinal dysfunction in sTBI patients. Reduced IGF-1 levels may impair intestinal mucosal barrier function and exacerbate gastrointestinal dysfunction, suggesting its potential as a biomarker for early identification of at-risk patients. Future research could explore therapeutic strategies involving IGF-1 supplementation to improve gastrointestinal outcomes in sTBI patients.

### Limitations and future perspectives

While this study provides valuable insights into the risk factors for gastrointestinal dysfunction in sTBI patients, some limitations must be acknowledged. First, the study is limited by its sample size, which may affect the generalizability of the findings. Larger, multi-center studies are needed to validate the predictive value of ICP, FC, and IGF-1 in diverse patient populations and settings. In addition, the study design was observational, which limits the ability to establish causality between the identified risk factors and gastrointestinal dysfunction. Future studies employing interventional designs could provide more robust evidence regarding the mechanisms and clinical significance of these factors.

Second, while the study focused on three key biomarkers (ICP, FC, and IGF-1) other potential contributors to gastrointestinal dysfunction, such as inflammatory cytokines, gut microbiota alterations, and nutritional status, were not explored. Incorporating these variables into future research could provide a more comprehensive understanding of the pathophysiology of gastrointestinal dysfunction in sTBI patients.

Third, the study did not evaluate the impact of therapeutic interventions targeting these risk factors on patient outcomes. For example, strategies to control ICP, reduce intestinal inflammation, or increase IGF-1 levels could potentially improve gastrointestinal function and overall prognosis in sTBI patients. Future clinical trials should investigate the efficacy of such interventions and their role in reducing morbidity and mortality.

From a future perspective, advancements in biomarker technology and personalized medicine could enable more precise prediction and management of gastrointestinal dysfunction in sTBI patients. Non-invasive monitoring tools, such as fecal biomarker panels, could offer early warnings of intestinal complications and guide timely interventions. In addition, understanding the relationship between the brain-gut axis and systemic inflammatory responses may open new therapeutic opportunities to mitigate gastrointestinal dysfunction and improve recovery after sTBI.

## Conclusion

The findings of this study highlight the importance of postoperative ICP, FC, and IGF-1 as independent risk factors for gastrointestinal dysfunction in sTBI patients. These indicators not only provide insights into the pathophysiological mechanisms underlying gastrointestinal dysfunction but also provide potential predictive value for early clinical intervention. Active management of ICP, monitoring of FC levels, and consideration of IGF-1 supplementation may help mitigate gastrointestinal complications and improve patient outcomes. Further research is needed to explore targeted therapies and refine predictive models for gastrointestinal dysfunction in sTBI patients.

## Data Availability

The original contributions presented in the study are included in the article/supplementary material, further inquiries can be directed to the corresponding author.
